# New Diagnostic Real-Time PCR for Specific Detection of *Mycoplasma hominis* DNA

**DOI:** 10.1155/2010/317512

**Published:** 2010-07-25

**Authors:** Andres Pascual, Katia Jaton, Béatrice Ninet, Jacques Bille, Gilbert Greub

**Affiliations:** ^1^Infectious Diseases Service, Department of Internal Medicine, University Hospital Center and University of Lausanne, 1011 Lausanne, Switzerland; ^2^Institute of Microbiology, University Hospital Center and University of Lausanne, 1011 Lausanne, Switzerland; ^3^Laboratory of Microbiology, University Hospital of Geneva, Geneva, Switzerland

## Abstract

*Mycoplasma hominis* is a fastidious micro-organism causing genital and extragenital infections. We developed a specific real-time PCR that exhibits high sensitivity and low intrarun and interrun variabilities. When applied to clinical samples, this quantitative PCR allowed to confirm the role of *M. hominis* in three patients with severe extragenital infections.

*Mycoplasma hominis *are fastidious bacteria, which are not detected on routinely used axenic media [1]. Lack of a cell wall makes these organisms naturally resistant to *β*-lactam antibiotics and not detectable by Gram staining. *M. hominis* is a recognized agent of genital infections in adults as well as of neonatal infections [2, 3]. Furthermore, it has been reported as the etiologic agent of various serious extra-genital infections such as brain abscess, pneumonia, mediastinitis, pericarditis, endocarditis, osteitis, arthritis, wound infections, peritonitis, and pyelonephritis both in immunosuppressed and in immunocompetent individuals [4–11]. Since most commonly used antibiotics in clinical practice are not active against *M. hominis,* the diagnosis of infections due to this fastidious bacterium is a crucial issue, especially for extra-genital cases.

In this context, we developed a real-time PCR assay for the detection of *M. hominis. *Then, this new PCR was applied to clinical samples obtained from patients suffering from extra-genital *M. hominis* infections. 

A forward primer MhF (5-TTTGGTCAAGTCCTGCAACGA-3′, position 2472–2493 of GenBank sequence AF443616), a reverse primer MhR (5′-CCCCACCTTCCTCCCAGTTA-3′, position 2553–2572 of AF443616), which amplifies a 101 bp part of the 16S rRNA-encoding gene, and a minor-groove binder probe labeled with 5′ VIC (TACTAACATTAAGTTGAGGACTCTA, position 2513–2537 of AF443616) were selected using the Primer Express software (Applied Biosystems, Darmstadt, Germany). The reactions were performed in a final volume of 20 *μ*l, including 0.2 *μ*M of each primer, 0.2. *μ*M of probe, 10 *μ*l 2× TaqMan Universal Master Mix (Applied Biosystems), and 5 *μ*l of DNA sample. The cycling conditions were 2 min at 50°C and 10 min at 95°C, followed by 45 cycles of 15 s at 95°C and 1 min at 60°C. An ABI Prism 7900 instrument (Applied Biosystems) was used for the amplification and detection of the PCR products. The specificity of the real-time PCR was high, being increased by the use of a TaqMan probe. No cross-amplification was observed when 5 ng of genomic DNA of humans, fungus (*Candida albicans *ATCC 10231), and 15 different bacterial strains were tested (*Mycoplasma pneumoniae, Ureaplasma parvum, Ureaplasma urealyticum, Gardnerella vaginalis, Lactobacillus *sp.*, Chlamydia trachomatis, Chlamydophila pneumoniae*, *Enterococcus faecalis, Escherichia coli, Haemophilus influenzae, Klebsiella pneumoniae, Pseudomonas aeruginosa, Staphylococcus saprophyticus, Streptococcus pyogenes,* and* Streptococcus agalactiae)*. A plasmid containing the target gene was constructed to perform the quantification step, as described previously [12].

Duplicates of 10-fold serial dilutions of the plasmid were run in ten independent experiments in order to analyze the sensitivity and the reproducibility of the PCR results. As shown in Figure 1(a), the intrarun reproducibility was excellent. As expected, intrarun variability was slightly higher at very low concentration of target DNA (Figure 1(b)). The interrun variability was low, except at a low concentration of 10 plasmid copies per 5 *μ*l (Figure 1(c)). Thus, the mean Ct values +/−1 standard deviation were of 29.64 +/− 0.38 (1.28%), 32.69 +/− 0.36 (1.10%), and 36.43 +/− 0.49 (1.34%) for 1000, 100, and 10 DNA copies per 5 *μ*l. The analytical sensitivity of the real-time PCR was 10 copies of plasmid control DNA per reaction mixture, that is, similar to the light-cycler PCR already reported by Baczynska et al. [14]. This sensitivity is about 10-fold better than the 16SrRNA broad-range PCR developed by Bosshard et al. [15]. When testing genomic DNA obtained by growing *M. hominis *in culture, we again obtained an excellent sensitivity which was of about 1000 bacteria/ml. When serially diluting a clinical sample (inguinal abscess of patient 1), the eubacterial PCR was slightly positive when the real-time specific *Mycoplasma *PCR was positive with a Ct of 33.4 (45 copies in 5 *μ*l of DNA) whereas eubacterial PCR was negative when the real-time specific *Mycoplasma *PCR was positive with a Ct of 36.3 (6.5 copies in 5 *μ*l of DNA). 

Since growing evidence supports the role of *M. hominis* as an emerging agent of extra-genital infections, the real-time PCR was tested in 34 clinical samples taken from 15 patients suffering from various extra-genital infections. These samples included physiologically sterile sites such as pleural fluid, percardial fluid, cerebrospinal fluid, and cardiac valve (*n* = 19) and from samples potentially contaminated with oropharyngeal flora (*n* = 15), that is, mainly respiratory tract samples (*n* = 8). DNA was extracted from 200 *μ*l of samples using the MagNAPureLC automated system (Roche) and the MagnaPureLC DNA isolation kit 1 (Roche). DNA was eluted in a final volume of 100 *μ*l of the elution buffer provided in the kit. A negative extraction control (DNA free water extracted in parallel of the specimen) was tested for each extraction run. Each sample was amplified in duplicate. Inhibition control (specimen spiked with 1 *μ*l containing 200 plasmid copies) and negative PCR mixture control were systematically tested. All samples were investigated because of clinical suspicion except two, for which the presence of *M. hominis* DNA was already detected by a broad spectrum 16SrRNA PCR directly performed on the specimens [16]. Both were confirmed positive by the new PCR with very high bacterial load (Table 1). DNA of *M. hominis* was also found in samples of 1 out of the 13 remaining patients.

For all 3 positive patients, DNA of *M. hominis *was detected in ≥3 specimens (Table 1). Moreover, for each of these 3 patients, at least one of the positive molecular results was further confirmed by culture with a commercial media (*Mycoplasma* IST, bioMérieux, France) or on blood agar (thin layer of bacteria and sequencing for identification) [13]. Due to the high level of conservation of the 16S rRNA, our real-time PCR is likely amplifying any member of the *M. hominis *group that includes *M. salivarium, M. orale,* and *M. arginii. *All cases presented in this paper were *M. hominis *sensu stricto as confirmed by sequencing 16S rRNA encoding gene. The clinical presentation of these three cases is summarized in Table 1. 

In conclusion, we developed a new PCR that exhibited high sensitivity and that allowed us to diagnose or confirm three extra-genital cases of *M. hominis* infections, which may remain undetected due to the fastidious growth of this bacterium. Recently, Masalma et al., using a cloning and/or pyrosequencing sequencing approach, also identified two extragenital *M. hominis * cerebral infections [4]. The advantage of their strategy is the broad spectrum of the approach that is not limited to *Mycoplasma *whereas the advantages of our real-time PCR are simplicity, rapidity, and lower risk of contamination. 

This new real-time PCR represents an efficient tool for the diagnosis of *M. hominis* infections that may contribute to better defining of the prevalence and pathogenicity of *M. hominis*. Moreover, it could help to improve clinical outcomes of severe extra-genital infections in patients not responding to commonly used beta-lactam antibiotics.

## Figures and Tables

**Figure 1:**
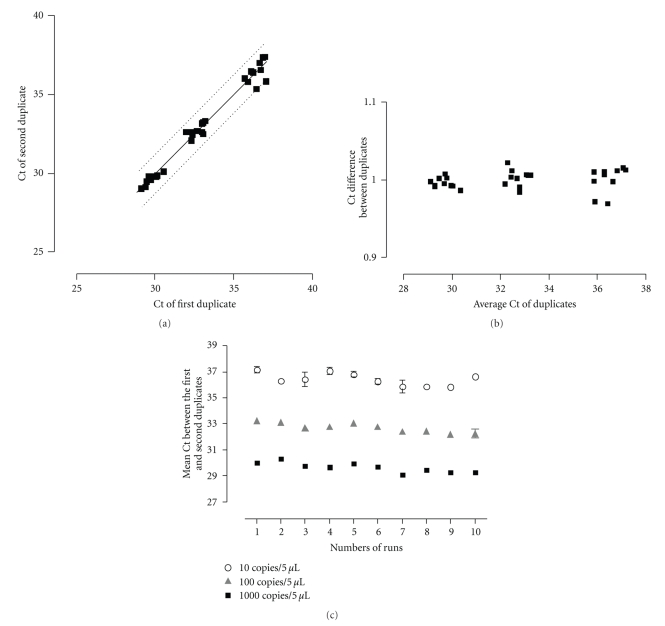
Intra and interrun reproducibility of the real-time PCR assessed on duplicate of plasmid positive controls performed at 10-fold dilutions from 1000 to 10 plasmid copies/5 *μ*L during 10 successive runs. (a) Plots of the cycle threshold (Ct) of first and second duplicates, showing intrarun variability of the real-time PCR between duplicates of positive plasmid controls; 95% confidence interval is shown by the dashed lines. (b) Bland-Altman graph showing the ratio of Ct of both duplicates according to the mean of the Ct of duplicates. (c) Plots of the mean of duplicate of plasmid positive controls according to each successive run, showing the low interrun variability of the real-time PCR. Standard deviations show the intrarun reproducibility of the PCR.

**Table 1:** Characteristics of three patients with *Mycoplasma hominis* extra-genital infections, including clinical presentation and results of the real-time PCR.

Patient no.	Age	Sex	Clinical infection	Underlying conditions	Other etiology	Clinical specimen	*M. hominis* qPCR results in copies/mL	Other positive diagnostic tests
1	54	Female	Inguinal lymphadenitis with abscess formation	Neutropenia, HIV	None	*Abscess fluid* ^a^	**16,175,000**	Specific culture^c^ and broad-spectrum PCR
						Cervix swab	4,400	
						Vaginal swab	130	
						Urine	82	

2	15	Female	Pneumonia and pericarditis	None	None	*Abscess fluid* ^a^	**98,500,000**	Specific culture^c^ and broad-spectrum PCR
						Pleural fluid	380,000	
						Pericardiac fluid	85	
						Sputum^b^	6	
						Pleural fluid^b^	0	

3	56	Male	Mediastinitis	Type A aortic dissection	None	*Mediastinal fluid* ^a^	**7,000**	Blood agar ^d^
						Mediastinal swab	460	
						**Blood**	5	

^a^ First samples documenting the infection;

^b^ Samples taken a few days after treatment start;

^c^Culture media: Mycoplasma IST, bioMérieux, France;

^d^ Conventional culture positive on blood agar plate followed by sequencing for identification [13].
